# Exome sequencing identifies a *KRT9* pathogenic variant in a Chinese pedigree with epidermolytic palmoplantar keratoderma

**DOI:** 10.1002/mgg3.703

**Published:** 2019-05-09

**Authors:** Changxing Li, Pingjiao Chen, Silong Sun, Kang Zeng, Jingyao Liang, Qi Wang, Sanquan Zhang, Meinian Xu, Zhijia Li, Xibao Zhang

**Affiliations:** ^1^ Department of Dermatology Nanfang Hospital, Southern Medical University Guangzhou China; ^2^ BGI Genomics, BGI‐Shenzhen Shenzhen China; ^3^ Department of Dermatology Guangzhou Institute of Dermatology Guangzhou China

**Keywords:** epidermolytic palmoplantar keratoderma, exome sequencing, Keratin 9

## Abstract

**Background:**

Epidermolytic palmoplantar keratoderma (EPPK) is a rare skin disorder and its pathogenesis and inheritability are unknown.

**Objective:**

To investigate the inheritance and pathogenesis of EPPK.

**Methods:**

Two EPPK cases occurred in a three‐generation Chinese family. Patient–parents trio EPPK was carried out and the identified candidate variants were confirmed by Sanger sequencing.

**Results:**

A heterozygous missense pathogenic variant, c.488G > A (p.Arg163Gln), in the keratin (*KRT*) 9 gene was detected in the proband and his son via targeted exome sequencing, and then validated by Sanger sequencing. This pathogenic variant cosegregated with the EPPK in extended family members, and was predicted to be pathogenic by SIFT, PolyPhen2, PROVEAN, and Mutation Taster. This heterozygous variation was not evident in 100 healthy controls.

**Conclusion:**

This report describes a *KRT9* c.488G > A (p.Arg163Gln) variant causing a diffuse phenotype of Chinese EPPK. The current results broaden the spectrum of *KRT9* pathogenic variants responsible for EPPK and have important implications for molecular diagnosis, treatment, and genetic counseling for this family.

**Abbreviations**
EPPK: Epidermolytic palmoplantar keratoderma
*KRT*: Keratin
NSVs: Nonsynonymous variants
PPK: palmoplantar keratodermas
SSMs: Splice acceptor‐site or donor‐site mutations

## 1 INTRODUCTION

Palmoplantar keratoderma (PPK) is a group of heterogenous genodermatoses characterized by hyperkeratotic skin in the palms and soles, clinically grouped into four patterns: diffuse, striate, focal, and punctate （Fukunaga, Kubo, Sasaki, Tsuruta, & Fukai, 2018; Knöbel, O'Toole, & Smith, 2015; Mao, Zhang, You, Xiao, & Zhao, 2018; Smith et al., 2018; Wang et al., 2016; Xiao et al., 2018）. Epidermolytic palmoplantar keratoderma (EPPK, OMIM144200) is a rare skin disorder with the main clinical feature of diffuse hyperkeratotic skin in the palms and soles (Wang et al., 2016; Xiao et al., 2018). Generally, no subjective symptoms accompany the disorder except mild itching. Histopathologic features of EPPK are dyskeratosis, epidermolytic hyperkeratosis, pronounced perinuclear vacuolization of the keratinocytes, and huge keratohyalin granules located in the upper stratum spinosum (Endo, Hatamochi, & Shinkai, 1997; Reis, Küster, Eckardt, & Sperling, 1992). Ultrastructurally, EPPK presents abnormal perinuclear tonofilament clumping and the presence of large and distorted keratohyalin granules (Szalai, Szalai, Becker, & Török, 1999). The pathogenesis of PPK remains elusive. Other types of PPK, disorders characterized by thickening of the skin on the palms of the hands and soles of the feet, can be inherited but some are acquired. The pathogenesis of this wide group of disorders involves structural proteins (keratins), cornified envelope (loricrin, transglutaminase), cohesion (plakophilin, desmoplakin, desmoglein1), cell‐to‐cell communication (connexins), and transmembrane signal transduction (cathepsin C) proteins (Chen et al., 2018; Li, Han, Han, Zeng, Zhang, & Ma, 2014; Li, Wen, et al., 2014). An autosomal dominant inheritance is usually seen in EPPK （Fukunaga et al., 2018; Knöbel et al., 2015; Mao et al., 2018; Smith et al., 2018; Wang et al., 2016; Xiao et al., 2018）. The genetic basis of EPPK has also been explored. Causal mutations have been detected in the *KRT1*, *KRT9*, and *KRT10* genes (Has & Technau‐Hafsi, 2016; Liang, Liu, Huang, & Zeng, 2014; Makino, Furuichi, Asano, & Shimizu, 2012; Terron‐Kwiatkowski et al.., 2006). We present our investigation of two cases with EPPK from a three‐generation Chinese family consisting of nine members, which suggested to us that the possibility of an autosomal dominant trait.

## 2 MATERIALS AND METHODS

### 2.1 Ethics statement

Human samples used in this study were obtained in accordance with the principles expressed in the Declaration of Helsinki, and were approved by the Institutional Review Board from Nanfang Hospital, Southern Medical University. Blood and skin biopsy sample collection procedures used were in accordance with the institutional and national ethical standards of human experimentation and all participants provided written informed consent.

### 2.2 Clinical material

**Figure 1:**
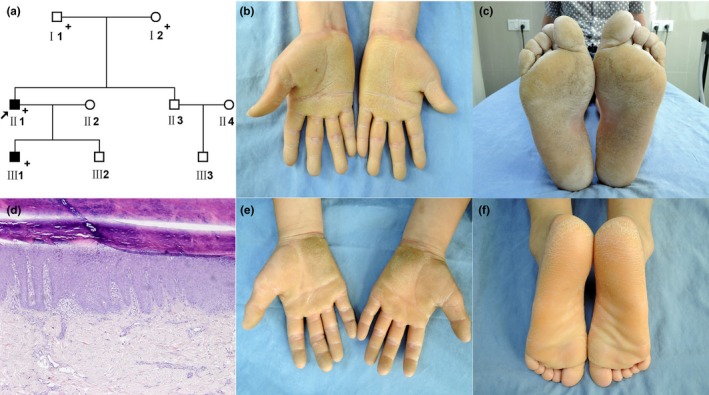
Family Pedigree and Clinical Phenotype of the Proband with EPPK. (a) Pedigree was constructed for the nine‐member family with EPPK. Squares and circles indicate males and females, respectively. Filled symbols indicate affected individuals. Arrow indicates the proband. “+” in pedigree indicates those who are subjected to exome sequencing. (b,c) Lesions of EPPK on proband. Hyperkeratosis yellow thickening plaques on the palms and soles. (d) Biopsy from the proband was hematoxylin and eosin (e,f) stained and visualized under light field microscopy (magnitude: ×100). Observed features included epidermal basket‐weave hyperkeratosis, acanthosis, a large number of keratohyalin granules in the upper part of the spinous cell layer, and superficial ectatic capillaries and perivascular infiltration with a few lymphocytes in the dermal layer

The proband (II1) (Figure 1a) was a 35‐year‐old man, who was presented with yellow thickening of the palms and soles with an erythematous margin for 35 years. At 1 month of age, he developed symmetrical erythematous on the palms and soles. At the age of 2 years, hyperkeratotic plaques appeared. Cutaneous examination showed hyperkeratosis yellow thickening plaques on the palms and soles (Figure 1b,c). Oral mucosa, teeth, and nails remained unchanged. The histological examination of the proband's lesion revealed epidermal basket‐weave hyperkeratosis, acanthosis, a large number of keratohyalin granules in the upper part of the spinous cell layer, and superficial ectatic capillaries and perivascular infiltration with a few lymphocytes in the dermal layer (Figure 1d). His elder (III1) (Figure 1a) son had similar symptoms on the palms and soles (Figure 1e,f). The electron microscopy revealed that the epidermis was acanthotic and thickened, and the compact stratum corneum. The stratum corneum was compact and regular distribution. The keratin filaments were remarkably clumped or aggregated and irregularly distributed in the stratum corneum.

### 2.3 Exome sequencing and sequence analysis

Exome sequencing was then performed. The data for this study were generated by Illumina Hiseq2500 (Illumina, USA). The sequence reads were aligned to the NCBI reference genome (hg19/GRCh37). We used various tools including Inheritance State Consistency Analysis (ISCA) for sequence alignment analysis, ANNOVAR for single‐nucleotide polymorphism (SNP) annotation, and GERP for substitution analysis to analyze the genome sequencing results. After annotation of variants, we focused only on nonsynonymous variants (NSVs), splice acceptor‐site or donor‐site mutations (SSMs), and insertions/deletions (indels) that were more likely to be pathogenic than other variants.

### 2.4 Validation of pathogenic variant by Sanger sequencing

The Sanger sequencing approach was used to validate the EPPK pathogenic variant in the *KRT9* gene. PCR was performed around the candidate pathogenic variant positions according to standard techniques. The PCR products were purified using QIAquick PCR purification kit from Qiagen (Germantown, MD) and sequenced by ABI Dye Terminator Cycle Sequencing on an ABI 3730XL DNA analyzer (Life Technologies, Carlsbad, CA). Sequence trace files were analyzed by Sequencer software version 4.5 (GeneCodes, Ann Arbor, MI).

## 3 RESULTS

### 3.1 Exome sequencing

Family members, I1, I2, Ⅱ1, and III1 underwent sequencing to examine a genetic cause for the disorder independently. An average of 10.8 billion bases of sequence was produced per individual as paired‐end, 90‐bp reads. After mapping to the human reference genome (NCBI Build 36.3, hg19), we achieved ~5.5 Gb of map able, targeted exome sequences with a mean coverage of 107.7‐fold. On average, 99.2% of the exomes were covered at least fourfold.

Under the assumption of autosomal dominant inheritance with full penetrance, heterozygous variants that were present in all the affected individuals, but not in unaffected participants, were selected as possible candidates. As a result, we detected 21 NSVs, SSMs, or indels in 20 candidate genes. We then used ANNOVAR and GERP to assess these variants for their probable functional impact. We focused on one heterozygous variant that we considered to be a likely candidate of the 21 possible variants. This was in *KRT9* (c.488G > A) located within chromosome 17. Therefore, we selected *KRT9* as a EPPK candidate gene for further studies.

### 3.2 Sequencing KRT9

**Figure 2:**
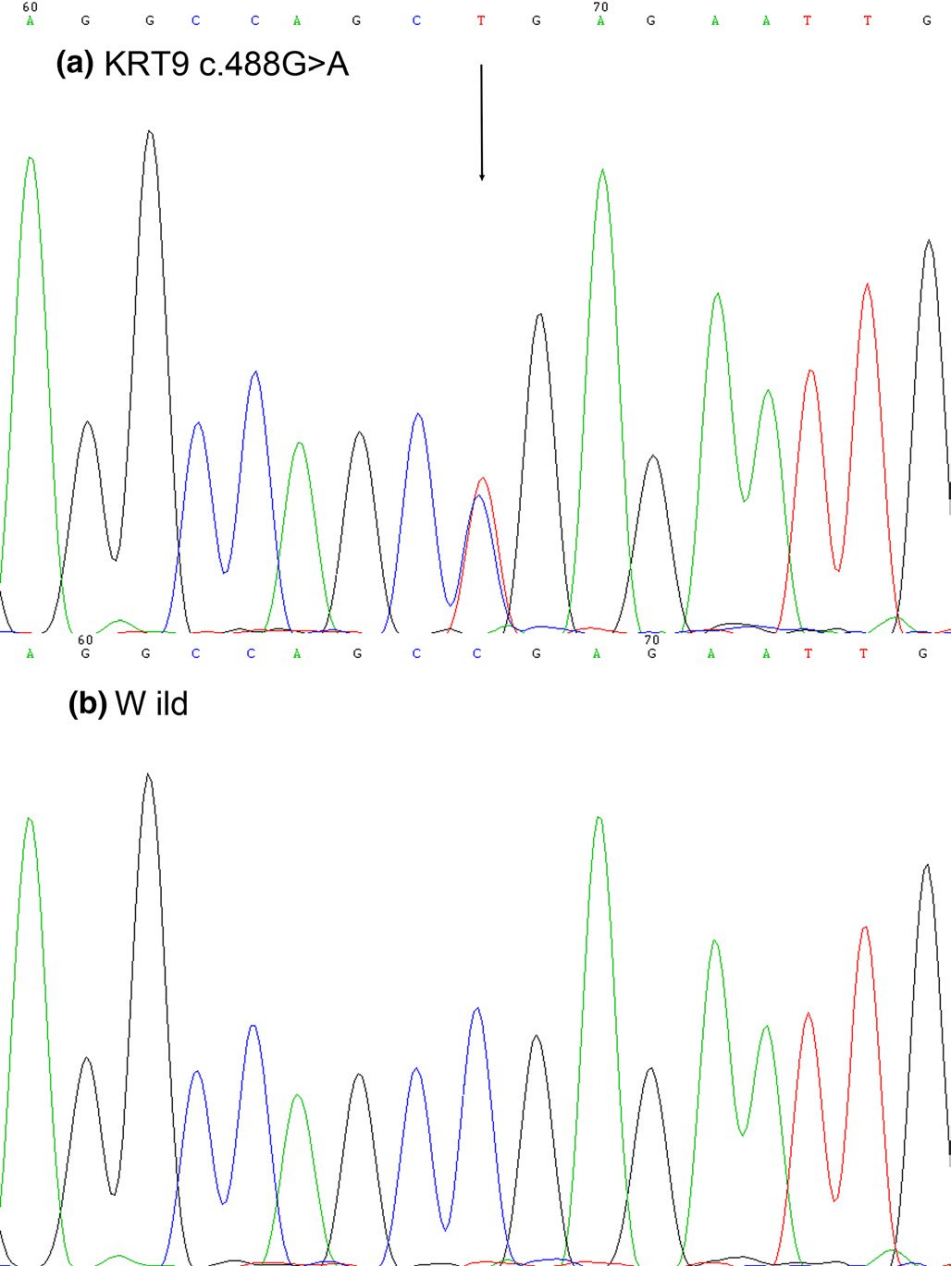
Mutant sequence in the *KRT9* gene. (a) Sanger sequencing showed a *KRT9* (c.488G > A) heterozygous mutationin the affected individuals (Ⅱ:1 and III:1) in the pedigree. (b) While other members of family showed wild‐type sequences

To investigate whether the pathogenic variant was inherited, all nine family members underwent sequencing of *KRT9*. A missense mutation encoding a c.488G > A alteration (p.Arg163Gln) in the first exon of the *KRT9* was found in two affected individuals (Figure 2a), and was absent in the seven unaffected family members (Figure 2b). We first tested for cosegregation of the pathogenic variant (c.488G > A) with the EPPK phenotype in the extended pedigree of the family. We further sequenced *KRT9* (c.488G > A) in 100 healthy controls and did not find a *KRT9* pathogenic variant.

## 4 DISCUSSION

PPK comprise a diverse group of acquired and hereditary disorders marked by excessive thickening of the epidermis of palms and soles (Has & Technau‐Hafsi, 2016). While PPKs may represent the sole or dominant clinical feature, they may also be associated with other ectodermal defects or extracutaneous manifestations (Has & Technau‐Hafsi, 2016). The exact morphological evaluation of keratoderma—diffuse, striate, focal, and punctate—is diagnostically helpful (Austin, 2016; Guo, Shi, & Tan, 2014; Has & Technau‐Hafsi, 2016; Ke et al., 2014; Mao et al., 2018). The degree of severity may vary in the same family or among individuals bearing similar pathogenic variants. The proband and his son were presented with yellowish, compact hyperkeratoses, surrounded by erythematous margins, and cover the entire surface of the palms and soles. Histopathology of the proband showed epidermolytic hyperkeratosis. The clinical feature and histopathology confirmed to the diagnosis of EPPK. Early onset and positive family history of the proband and his son suggest a genetic cause. Previous studies showed that similar clinical pictures of PPK may result from pathogenic variants in different genes. Therefore, pathogenic variant analysis was required to determine the precise type of these patients. By exome sequencing, a heterozygous missense pathogenic variant, c.488G > A (p.Arg163Gln), in the *KRT9* gene was detected in the proband and his son, and then validated by Sanger sequencing. Usually caused by pathogenic variants in the *KRT9* gene, this is the most common form of EPPK. *KRT9*c.488G > A (p.Arg163Gln) pathogenic variant showed autosomal dominant inheritance and perfectly cosegregated with the EPPK phenotype in all family members. This heterozygous variation was not evident in the proband's parents and 100 healthy controls, which suggested that *KRT9* p.Arg163Gln pathogenic variant was a de novo pathogenic variant in the proband and the pathogenic variant of his elder son was inherited from the proband. Keratin 9, expressed in suprabasal cells of the epidermis of palm and sole, where it contributes to reinforcing the skin in these regions to greater stress, is the source of several pathogenic variants causing EPPK (Austin et al., 2016).

In conclusion, we have identified a pathogenic variant in *KRT9* gene in two patients from one family. Our finding expands the mutant spectrum of *KRT9* gene. Identification of more individuals carrying new variants in *KRT9* gene is needed to fully characterize the full phenotypic spectrum associated with EPPK.

## IRB STATEMENT

Human samples used in this study were obtained in accordance with the principles expressed in the Declaration of Helsinki, and were approved by the Institutional Review Board from Nanfang Hospital, Southern Medical University. Blood and skin biopsy sample collection procedures used were in accordance with institutional and national ethical standards of human experimentation and all participants provided written informed consent.

## COMPETING INTERESTS

The authors state no conflict of interest.

## References

[mgg3703-bib-0001] Austin, S. W. , Cope, A. , Fernandez, M. , & Parekh, P. (2016). Infantile epidermolytic ichthyosis with prominent maternal palmoplantar keratoderma. Dermatology Online Journal, 22(4). pii: 13030/qt96w8m091. 27617465

[mgg3703-bib-0002] Chen, P. , Sun, S. , Zeng, K. , Li, C. , Wen, J. , Liang, J. , … Zhang, X. (2018). Exome sequencing identifies a TCF4 mutation in a Chinese pedigree with symmetrical acral keratoderma. Journal of the European Academy of Dermatology and Venereology, 32(7), 1204–1208. 10.1111/jdv.14591 28921696

[mgg3703-bib-0003] Endo, H. , Hatamochi, A. , & Shinkai, H. (1997). A novel mutation of a leucine residue in coil 1A of keratin 9 inepidermolytic palmoplantar keratoderma. The Journal of Investigative Dermatology, 109(1), 113–115. 9204965 10.1111/1523-1747.ep12276751

[mgg3703-bib-0004] Fukunaga, Y. , Kubo, A. , Sasaki, T. , Tsuruta, D. , & Fukai, K. (2018). Novel KRT9 missense mutation in a Japanese case of epidermolytic palmoplantar keratoderma. Journal of Dermatology, 45(4), e72–e73. 10.1111/1346-8138.14115 29068086

[mgg3703-bib-0005] Guo, Y. , Shi, M. , Tan, Z. P. , & Shi, X. L. (2014). Possible anticipation in familial epidermolytic palmoplantar keratoderma with the p. R163W mutation of Keratin 9. Genetics and Molecular Research, 13(4), 8089–8093. 10.4238/2014.October.7.3 25299193

[mgg3703-bib-0006] Has, C. , & Technau‐Hafsi, K. (2016). Palmoplantar keratodermas:Clinical and genetic aspects. Journal Der Deutschen Dermatologischen Gesellschaft, 14(2), 123–139. quiz 140. 10.1111/ddg.12930 26819106

[mgg3703-bib-0007] Ke, H. P. , Jiang, H. L. , Lv, Y. S. , Huang, Y. Z. , Liu, R. R. , Chen, X. L. , … Zhang, X. N. (2014). KRT9 gene mutation as a reliable indicator in the prenatal molecular diagnosis of epidermolytic palmoplantar keratoderma. Gene, 546(1), 124–128. 10.1016/j.gene.2014.05.048 24862219

[mgg3703-bib-0008] Knöbel, M. , O’Toole, E. A. , & Smith, F. J. (2015). Keratins and skin disease. Cell and Tissue Research, 360(3), 583–589. 10.1007/s00441-014-2105-4 25620412

[mgg3703-bib-0009] Li, C. X. , Han, C. L. , Zeng, K. , Zhang, X. B. , & Ma, Z. L. (2014). Clinical, demographic and histopathological features of symmetrical acral keratoderma. British Journal of Dermatology, 170(4), 948–951. 10.1111/bjd.12754 24341804

[mgg3703-bib-0010] Li, C. X. , Wen, J. , Zeng, K. , Tian, X. , Li, X. M. , & Zhang, X. B. (2014). Ultrastructural study of symmetrical acral keratoderma. Ultrastructural Pathology, 38(6), 420–424. 10.3109/01913123.2014.930080 24956169

[mgg3703-bib-0011] Liang, Y. H. , Liu, Q. X. , Huang, L. , & Zeng, K. (2014). A recurrent p. M157Rmutation of keratin 9 gene in a Chinese family with epidermolytic palmoplantar keratoderma and literature review. International Journal of Dermatology, 53(8), e375–379. 10.1111/ijd.12352 24899405

[mgg3703-bib-0012] Makino, T. , Furuichi, M. , Asano, Y. , & Shimizu, T. (2012). Novel mutation of the KRT 10 gene in a Japanese patient with epidermolytic hyperkeratosis. Journal of Dermatology, 39(1), 87–89. 10.1111/j.1346-8138.2011.01234.x 21463361

[mgg3703-bib-0013] Mao, B. , Zhang, J. , You, Y. , Xiao, J. , & Zhao, X. (2018). Mutations in the highly conserved 1A rod domain of keratin 9 responsible for epidermolytic palmoplantar keratoderma in four Chinese families. Journal of Dermatology, 45(2), e45–e46. 10.1111/1346-8138.14087 29044727

[mgg3703-bib-0014] Reis, A. , Küster, W. , Eckardt, R. , & Sperling, K. (1992). Mapping of a gene for epidermolytic palmoplantar keratoderma to the region of the acidic keratin gene cluster at 17q12‐q21. Human Genetics, 90(1–2), 113–116. 1385292 10.1007/BF00210752

[mgg3703-bib-0015] Smith, F. J. D. , Kreuser‐Genis, I. M. , Jury, C. S. , Wilson, N. J. , Terron‐Kwiatowski, A. , & Zamiri, M. (2018). Novel and recurrent mutations in keratin 1 cause epidermolytic ichthyosis and palmoplantar keratoderma. Clinical and Experimental Dermatology. 10.1111/ced.13800 PMC7116359 30288772

[mgg3703-bib-0016] Szalai, S. , Szalai, C. , Becker, K. , & Török, E. (1999). Keratin 9 mutations in the coil 1A region in epidermolytic palmoplantar keratoderma. Pediatric Dermatology, 16(6), 430–435. 10.1046/j.1525-1470.1999.00111.x 10632938

[mgg3703-bib-0017] Terron‐Kwiatkowski, A. , van Steensel, M. A. , van Geel, M. , Lane, E. B. , McLean, W. H. , & Steijlen, P. M. (2006). Mutation S233L in the 1B domain of keratin 1 causes epidermolytic palmoplantar keratoderma with "tonotubular" keratin. The Journal of Investigative Dermatology, 126(3), 607–613. 10.1038/sj.jid.5700152 16439967

[mgg3703-bib-0018] Wang, P. , Kang, X.‐J. , Tang, X.‐H. , Liu, J.‐Y. , Li, W.‐Z. , Wang, W.‐J. , … Chen, W.‐J. (2016). Six generations of epidermolytic palmoplantar keratoderma, associated with a KRT9 R163W mutation. Cancer Genetics, 209(11), 515–524. 10.1016/j.cancergen.2016.10.002 27864007

[mgg3703-bib-0019] Xiao, H. , Guo, Y. , Yi, J. , Xia, H. , Xu, H. , Yuan, L. , … Deng, H. (2018). Identification of a novel keratin 9 missense mutation in a Chinese family with epidermolytic palmoplantar keratoderma. Cellular Physiology and Biochemistry, 46(5), 1919–1929. 10.1159/000489381. 29719290

